# Macrophage-Derived Extracellular Vesicles as Carriers of Alarmins and Their Potential Involvement in Bone Homeostasis

**DOI:** 10.3389/fimmu.2019.01901

**Published:** 2019-08-08

**Authors:** Bartijn C. H. Pieters, Alfredo Cappariello, Martijn H. J. van den Bosch, Peter L. E. M. van Lent, Anna Teti, Fons A. J. van de Loo

**Affiliations:** ^1^Experimental Rheumatology, Radboud University Medical Center, Nijmegen, Netherlands; ^2^Research Laboratories - Department of Oncohematology IRCCS Bambino Gesù Children's Hospital, Rome, Italy; ^3^Department of Biotechnological and Applied Clinical Sciences, University of L'Aquila, L'Aquila, Italy

**Keywords:** alarmins, bone homeostasis, extracellular vesicles, exosomes, macrophages

## Abstract

Extracellular vesicles are a heterogeneous group of cell-derived membranous structures, which facilitate intercellular communication. Recent studies have highlighted the importance of extracellular vesicles in bone homeostasis, as mediators of crosstalk between different bone-resident cells. Osteoblasts and osteoclasts are capable of releasing various types of extracellular vesicles that promote both osteogenesis, as well as, osteoclastogenesis, maintaining bone homeostasis. However, the contribution of immune cell-derived extracellular vesicles in bone homeostasis remains largely unknown. Recent proteomic studies showed that alarmins are abundantly present in/on macrophage-derived EVs. In this review we will describe these alarmins in the context of bone matrix regulation and discuss the potential contribution macrophage-derived EVs may have in this process.

## Introduction

Intercellular communication is an important biological process which allows cells to coordinate their response to physiological changes, environmental triggers and pathogenic invaders in a spatial and temporal fashion. A new addition to the intercellular communication system are extracellular vesicles (EVs) (1). EVs are small cell membrane-derived phospholipid bilayer structures that range in diameter from 30 to 2000 nm. Previously, they were considered to be merely cellular waste products, nowadays EVs are recognized as regulatory structures, produced and released by an actively regulated intracellular and energy dependent process as a means to shuttle complex cargo and deliver biological information to recipient cell/tissues. A distinction can be made between three different subtypes of vesicles based on their biogenesis and size: exosomes (30–150 nm diameter) released by exocytosis, microvesicles or microparticles (100–1,500 nm diameter) formed by budding from the plasma membrane (shedding vesicles, matrix vesicles) and apoptotic bodies (500–2,000 nm diameter) released from apoptotic cells (2). While the latter are the specific products of the complex processes of cells undergoing programmed cell death, all other EV subtypes are not phenotypically linked to cell death.

Upon release, EVs can interact with recipient cells in a number of ways. Host receptor activation can be induced via the interaction of vesicle membrane proteins either in a juxtacrine fashion or by paracrine signaling after being cleaved and released from EVs. EVs can also fuse with the cell membrane, mediating membrane receptor transfer and releasing its cargo intracellularly. Finally, EVs can be taken up by cells via endocytosis, delivering their cargo inside endocytic vacuoles (3). EV-mediated transfer of protein, genetic information (DNA, RNA and predominately small non-coding RNA and microRNA) is shown to be very efficient and intravesicular cargo is protected from degradation in the intercellular environment by their lipid bilayer membrane (4).

The involvement of EVs in bone homeostasis was previously thought to be primarily via matrix vesicles, a specific subgroup of EVs that consist of small membrane particles (20–200 nm), which bud off from the plasma membrane of mineralizing cells, such as osteoblasts and chondrocytes, prior to the onset of matrix mineralization [reviewed in (5)]. Ultrastructural studies in the late 1960's have shown that cartilage calcification starts in and around matrix vesicles, and matrix vesicles have since been implicated to play a role in the calcification of bone, cartilage, and dentin. However, more recent studies show the importance of bone cell-derived EVs as mediators of intercellular communication and their function in bone homeostasis and remodeling [reviewed in (6)]. In this review we will briefly summarize the communication between bone cells via EVs and thereafter focus on the potential role of macrophage-derived EVs carrying alarmins as contributors of bone remodeling.

## The Function of EVs in Bone Remodeling Extends That of Being Matrix Particles

The skeleton physiology is not exempt from the participation of EVs in biological processes. In fact, the skeleton houses a complex microenvironment that hosts a great diversity of cells, such as osteoblasts, osteoclasts, osteocytes, and other myeloid cells of the bone marrow, including macrophages. All of these cells are known to release EVs which can regulate each other's function.

### Osteoblasts

Osteoblasts are specialized mesenchymal cells that are responsible for bone matrix synthesis and mineralization during both initial bone formation and later bone remodeling. These cells were first recognized to promote mineralization, releasing matrix vesicles able to initiate nucleation of hydroxyapatite crystals (7). Across the years, further investigations deeply characterized the cargo and functions of matrix vesicles derived from osteoblasts and osteoblast-like cells, identifying alkaline phosphatase, the pyrophosphate generating enzyme PC1 and the pyrophosphate channel ANK as key mechanisms causing pyrophosphate production and influx into these EVs by hydroxyapatite nucleation.

Primary osteoblasts and their EVs share a similar gene profile, which included the expression of *atf4, alp, runx2, osx, col1a1* (8). A deeper proteomic characterization of exosomes derived from osteoblastic cell line (MC3T3) revealed many proteins related to the osteogenic pathways, such as mTOR, integrins, and eukaryotic initiation factor-2 signaling (9). Transcriptomic profiling performed in mineralizing MC3T3 revealed EVs containing osteogenic miRNAs (10). Exposure of mouse bone marrow-derived stromal ST2 cells to MC3T3-derived EVs, induced their osteogenic differentiation, manifested by the up-regulation of osteogenic markers, such as runt-related transcription factor 2 (RUNX2) and alkaline phosphatase, and enhancing matrix mineralization through the modulation of calcium, Wnt, insulin, and TGF-β signaling pathways.

van Leeuwen et al. studied the molecular profile of EVs from human osteoblasts, both in naïve and mineralizing conditions (11, 12). Comparing the cellular and EV mRNAs of osteoblasts, they showed that EVs were enriched with mRNAs related to protein translation, RNA processing and cell-to-cell communication, in particular with osteoclasts (NFKBIB, PGF), adipocytes (FGF1) and hematopoietic stem cells (FLT3LG, IL18) (11). Taken together, these findings suggest that osteoblasts release EVs capable of enhancing osteogenic differentiation, thereby contributing to bone formation and mineralization.

### Osteoclasts

Osteoclasts are unique in their ability to resorb bone and play an important role in bone turnover. A tight crosstalk with osteoblasts and osteocytes, which influence osteoclastogenesis by the factors they produce, is crucial to synchronize the activities in homeostatic bone remodeling (13, 14). Osteoclasts differentiate from myeloid progenitor cells under the influence of macrophage colony-stimulating factor (M-CSF) and receptor activator of nuclear factor kappa-B ligand (RANKL) (15, 16). Next to RANKL signaling, a co-stimulatory signal is required for osteoclastogenesis. After full differentiation, mature multinucleated osteoclasts can start to secrete acids and lytic enzymes that together resorb the bony tissues (14).

A recent study reported that osteoclast precursors are capable of releasing exosomes that could directly promote the osteogenic differentiation of the recipient mesenchymal stem cells (17). On the other hand, Liu and colleagues showed that mature osteoclast-derived exosomes were internalized by osteoblasts leading to a miR-214-3p-dependent inhibition of osteoblast activity and bone formation (18).

Interestingly, osteoclast EVs seemed to be involved in their own maturation. Holliday's group showed that pre-osteoclast EVs promoted osteoclastogenesis in whole bone marrow stromal cell cultures upon Vitamin D_3_ treatment, while mature osteoclast EVs inhibited osteoclastogenesis in the same culture conditions (19). This effect was demonstrated to be due to RANK expressed by EVs only from mature osteoclasts, presumably able to bind competitively RANKL in the microenvironment, similarly to osteoprotegerin (OPG). Furthermore, EVs from osteoclasts have been shown to transfer osteoclast-osteoblast coupling factors. RANK-expressing EVs from mature osteoclasts bind RANKL on osteoblasts, activating the reverse signaling and inducing RUNX2 activity in osteoblasts and bone formation (20). The idea of EV-based osteoclast-to-osteoblast coupling is strengthened by the paper of Sun et al. showing that EVs from osteoclasts express EphrinA2, which binds the Eph receptor expressed by osteoblasts, inhibiting bone formation (21, 22). These findings highlight the importance of EVs in the communication between osteoclasts and osteoblasts, but it is yet to be determined what the relative contribution of the vesicles is compared to the total secretome of these cells.

### Osteocytes

Osteocytes, the end stage of osteoblast differentiation, are matrix-embedded cells mainly involved in the regulation of bone remodeling and in the adaptation to mechanical forces (23). Morrel et al. found that mechanical stimulation activated osteocyte network inducing Ca^2+^-dependent contractions and enhancing the production and release of EVs containing RANKL, OPG and sclerostin (SOST) (24).

In comparison to osteoblast- and osteoclast-derived EVs much less is known about osteocyte-derived EVs. Osteocytes produce a unique EV population, described by Sato et al. in the osteocyte-ablated mouse model. They characterized circulating EVs of osteocyte-less mice and found 12 downregulated miRNAs in plasma. Furthermore, they described that this pool of miRNAs was enriched in EVs from osteocyte-like MLO-Y4 cells compared to non-osteocytic ST2 cells (25). Among the osteocyte miRNAs is miR-218 which could be taken up by osteoblasts, resulting in downregulation of SOST leading to osteogenic activity (26).

### Other Bone Interacting Cells

The balanced interplay between these three bone cell types is also under control of immune cells like macrophages and T-cells and often during inflammation the homeostatic situation is turned into accelerated bone loss. These immune cells produce cytokines that steer the differentiation of progenitor cells into osteoblasts or osteoclasts thereby influencing bone regeneration.

Interestingly, the biogenesis of EVs is controlled by intracellular Ca^2+^ concentrations in the EV-producing cells and, furthermore, EVs are often carriers of calcium ions contributing to calcification of tissues (27). As bone regulates calcium homeostasis in the body, it may indirectly influence EV biogenesis as well. Moreover, cytokines and growth factors can alter intracellular Ca^2+^ levels by depleting calcium from the endoplasmic reticulum and by increasing calcium influx from the extracellular space. Hence, inflammation can regulate both the extra- and intracellular Ca^2+^ levels and thereby regulate EV biogenesis although many other intracellular mechanisms are involved as well. In the next paragraphs we will discuss how EVs derived from innate immune cells might communicate with bone cells and regulate bone homeostasis via alarmins.

## Macrophages as a Source for EVs Carrying Alarmins

Macrophages are a highly heterogenous population derived from the myeloid linage that can reside in bone either as resident cells or as a result of recruited myeloid precursors, mainly monocytes, that differentiate in the tissue. The interplay between macrophages and bone cells is critical to bone formation and repair. Osteal macrophages, also known as osteomacs, are one of these resident macrophages located in close proximity to the bone surface and do not express TRAP (28). However, they colocalize with TRAP positive osteoclasts and are found immediately adjacent or near to giant osteoclasts at catabolic sites (29). Osteomacs are also tightly associated with osteoblasts in the endosteal and the periosteal surface. When osteoblasts undergo apoptosis they are phagocytosed together with the debris by neighboring osteomacs (28). Osteomacs have also been shown to support bone formation and osteoblast mineralization *in vitro* and *in vivo* using a mouse model in which macrophages were ablated (MaFIA, macrophage Fas-induced apoptosis mouse) (28).

Apart from being crucial in homeostasis of normal bone, macrophages also play a critical role in inflammation-driven bone diseases (30). Tissue damage elicited by external (injuries, chemicals, infection) and internal triggers (DNA damage, immunological reactions) or by shortage (nutrients, oxygen) or excess (sugar, cholesterol) of factors can induce macrophage activation that disturbs bone homeostasis and causes bone destruction. Most of the damage associated factors are first sensed by resident macrophages that become stressed and upon activation recruit more macrophages. Resident and recruited macrophages respond to their local environment and activate specific transcriptional programs that drive them to a spectrum of different phenotypes ranging from pro-inflammatory M-1 like to anti-inflammatory M-2 like macrophages (31). When macrophages become stressed, pro- and anti-inflammatory mediators are released into the micro milieu that regulate innate and adaptive immune cells and may cause disbalanced bone homeostasis (32).

The majority of pro-inflammatory factors that are released by macrophages are rapidly suppressed by many feedback mechanisms. However, EVs are able to deliver pro-inflammatory factors to other cells in a protected way (33). Nevertheless, not all secreted proteins detected in the medium are also present in the EVs since packaging of the biomolecular cargo within the macrophage EVs is a regulated process (34). LPS stimulated macrophages release interleukins in the medium that were absent in their exosomes (35). On the other hand, many alarmins, damage-associated molecular patterns (DAMPs), are present in macrophage-derived EVs. For example, New et al. showed that macrophage-derived EVs are enriched in S100A9 and Anx5 and contribute to microcalcifications observed in atherosclerotic plagues (36). Using gain- and loss-of-function experiments the authors reveal the critical role for Anx5-S100A9 complexes in this process, highlighting the functional activity of EV-carried alarmins.

### Alarmins

Alarmins are endogenous molecules that are constitutively available and released upon cellular stress and activate the immune system, causing inflammation *in vivo*. Many alarmins are intracellular proteins that are both passively and actively secreted. Passive release is often associated with cell injury or death, whereas active release is regulated by mechanisms independent of ER-Golgi routes, such as degranulation or pyroptosis. Upon release, alarmins can bind a range of receptors among which toll-like receptors (TLRs) and receptors for advanced glycosylation end products (RAGE) are the most studied (37).

Proteomic studies on both monocyte- and macrophage-derived EVs show the presence of a large variety of alarmins, including annexins, galectins, heat-shock proteins and S100-alarmins (38–47). An overview of EV-associated alarmins is presented in Table 1. Within these studies different EV-populations were studied, ranging from exosomes to microparticles. Differential ultracentrifugation protocols were used for most studies, either in combination with density gradient or precipitation techniques. Most alarmins were present in the majority of the studies, including HSP-90, annexins, and several S100-proteins. Simultaneously, part of the alarmins were only found in a limited number of studies. These observed differences could be due to the different isolation methods, EV-subtypes investigated or sensitivity of the proteomic analysis.

**Table 1:** List of alarmins found by proteomic analysis of monocyte and macrophage-derived EVs.

	**Vesicle subtype**	**References**
**Heat-shock proteins**		
Heat shock conjugate 71 kDa	Exosomes, microparticle, microvesicles	(38, 39, 41, 42, 44–47)
HSP-β1	Exosomes, microparticle	(38, 39, 43–45, 47)
HSP-70 (protein 1A)	Exosomes, microparticle	(38, 39, 41, 43–45, 47)
HSP-70 (protein 4)	Exosomes, microparticle	(39–42, 44, 45, 47)
HSP-70 (protein 13)	Exosomes	(39, 41, 42)
HSP-75, mitochondrial	Exosomes, microparticle	(39, 41, 43, 44, 47)
HSP-90α	Exosomes, microparticle, microvesicles	(38–42, 44–47)
HSP-90β	Exosomes, microparticle, microvesicles	(38–47)
HSP-105	Exosomes, microparticle	(39, 41–47)
10 kDa heat shock protein	Exosomes, microparticle	(38, 39, 41, 42, 44, 45, 47)
60 kDa heat shock protein	Exosomes, microparticle	(39, 41–47)
**Annexins**		
Annexin A1	Exosomes, microparticle	(38–45, 47)
Annexin A2	Exosomes, microparticle	(38–45, 47)
Annexin A3	Exosomes, microparticle	(39–42)
Annexin A4	Exosomes, microparticle	(38–43, 45, 47)
Annexin A5	Exosomes, microparticle, microvesicles	(38, 39, 41–47)
Annexin A6	Exosomes, microparticle, microvesicles	(38–41, 43–47)
Annexin A7	Exosomes, microparticle	(38, 39, 41–45, 47)
Annexin A11	Exosomes, microparticle	(38–41, 43–45, 47)
**Galectins**		
Galectins-1	Exosomes, microparticle	(38, 39, 41, 42, 45, 47)
Galectin-3	Exosomes, microparticle	(39, 41, 42, 45, 47)
Galectin-7	Exosomes, microparticle	(38, 39, 44, 47)
Galectin-9	Exosomes, microparticle	(38, 39, 47)
Galectin-9B	Exosomes, microparticle	(39, 45)
**S100-alarmins**		
S100-A4	Exosomes, microparticle	(38, 39, 41, 42, 45, 47)
S100-A6	Exosomes, microparticle	(38, 39, 41, 42, 45, 47)
S100-A8	Exosomes, microparticle	(38, 39, 45, 47)
S100-A9	Exosomes, microparticle	(39, 45, 47)
S100-A10	Exosomes, microparticle	(38, 39, 41, 42, 45, 47)
S100-A11	Exosomes, microparticle	(38, 39, 41, 42, 45, 47)
S100-P	Exosomes	(39)
**Miscellaneous**		
Cathelicidin	Exosomes	(39)
Defensin α3	Exosomes	(39)
Endoplasmin	Exosomes, microparticle	(38, 39, 41–45, 47)
Fibronectin	Exosomes, microparticle	(38, 39, 42, 43, 45–47)
HMGB1	Exosomes, microparticle	(41, 43, 45)
Nucleolin	Exosomes, microparticle	(39, 41–45, 47)
Thymosin β4	Exosomes, microparticle	(39, 42, 45)
78 kDa glycoseregulated protein	Exosomes	(42)

Recruitment of alarmins into EVs seems to be partially dependent on the macrophage activation state. Stimulation of macrophages with curdlan, a bacterial β-glucan, increased vesicle-mediated protein secretion, and specifically increased the amount of alarmins found in their EVs (38). Similarly, infection of macrophages with influenza A virus, resulted in an increase in alarmins found in their produced EVs (39). It remains to be investigated whether polarization of macrophages also changes alarmin expression of their EVs, it has however been shown that microvesicles produced by M1 and M2 macrophages contain different mRNAs that can identify the macrophage phenotype (48).

From these proteomic studies it is not possible to determine which of these alarmins are surface-accessible. As most receptors recognizing alarmins (TLRs, RAGE) sense the extracellular milieu it is important which of these alarmins are carried on the surface of EVs. A recent study by Cvjetkovic et al. presented a novel work-flow designed to identify proteins localized on the surface of EVs (49). Using a multiple proteomics approach, combining proteinase treatment and biotin tagging, they were able to identify many proteins of cytosolic origin that were localized on the surface of mast cell-derived EVs. Among the identified proteins were a number of alarmins, including nucleolin, S100-A9, -A10, -A13, galectin-1, and several heat shock proteins. Interestingly, all annexins (A1-A7, A11, and A13) were absent from the surface, and were instead present intravesicularly (49). In contrast, Stewart, et al. showed that annexin-2 is localized on the surface of EVs (50). These discrepancies could be due to the different cell types used and the different sub-groups of EVs investigated. Microvesicles seem to more predominant in their surface expression of annexin-V compared to exosomes, as demonstrated by Heijnen et al. (51). The sorting mechanism responsible for protein localization remains to be identified.

## The Effect of Alarmins on Osteoblasts and Osteoclasts

Alarmins play wide roles in different cell types. Multiple studies described the profound involvement of families of alarmins in osteocyte, osteoblast and osteoclast differentiation and function, including annexins, galactins, heat shock proteins, S100-proteins and various other proteins, although detailed studies for many of the individual family members are still lacking.

### Annexins

Annexins are autocrine/paracrine factors secreted by several cell types. Among them, Annexin 2 (AnxII) was demonstrated to increase bone resorption (52). This effect was shown to be due to activation of bone marrow stromal cells with the overexpression of GM-CSF and RANKL, both being pro-osteoclastogenic factors (53). Additionally, a previous study showed that overexpression of AnxII stimulated osteoclast formation (54). Another study poses that AnxII is only involved in the proliferation of osteoclast precursors, probably via stimulation of GM-CSF production, but not in the later multinucleation stages of osteoclast differentiation (52). The receptor that mediates these effects remains to be elucidated. However, most studies described an autocrine effect of osteoclast-produced AnxII, leaving the importance of macrophage-derived AnxII in the stimulation of osteoclasts unknown.

### Galectins

Galectins are a class of proteins that bind specifically to β-galactoside sugars, consisting of 15 members, of which 9 are known in humans and 11 in mice. These soluble proteins have both intra- and extracellular functions (55).

Galectin-1 (Gal-1) has been proposed to mediate cell-to-cell and cell-to-matrix adhesion (55). Furthermore, galectin-1 has been found both to promote and inhibit cell proliferation of a number of cells. In particular, Gal-1 was demonstrated to decrease differentiation of bone marrow stromal cells (56).

Galectin 3 (Gal-3) is another member of the galectin family found to affect both osteoblast and osteoclast differentiation. It has been demonstrated that exogenous recombinant Gal-3 inhibited terminal differentiation of a human pre-osteoblast cell line (57). Weilner et al. found that Gal-3 affected osteogenic differentiation of mesenchymal stem cells (MSCs) and, interestingly, that Gal-3 can be detected in EVs from plasma. Administration of Gal-3-EVs to MCSs increased osteoblastogenesis, preventing β-catenin degradation (58). On the other hand, Gal-3 strongly decreased osteoclast formation from precursors by suppressing nuclear factor of activated T-cells c1 (NFATc1), whereas Gal-3-deficient bone marrow cells had an increased osteoclastogenic potential. Moreover, addition to mature osteoclasts inhibited their resorptive capacity (59, 60). Likewise, Gal-9 markedly decreased osteoclast formation from cell lines and bone marrow cells, probably via binding to its receptor T-cell immunoglobin- and mucin-domain-containing molecule 3 (Tim-3) (61).

### Heat Shock Proteins

Heat shock proteins (HSPs) are among the most well-studied alarmins. Under physiological conditions they act as intracellular chaperone proteins, but some members are secreted upon stress. Stress factors such as IL-1β and TNFα have been shown to increase HSP60 secretion. These increased HSP60 levels were shown to promote osteoclast formation and activity *via* potentiation of RANK-RANKL signaling. The same study showed that this effect runs *via* binding of HSP60 to TLR2 (62). The finding that HSP60 is an agonist for the triggering receptor expressed in myeloid cells (TREM)2 receptor, which is part of the co-stimulatory signaling that is needed for osteoclastogenesis, might give an additional mechanism of how HSP60 might increase osteoclastogenesis (63).

In contrast, the HSP70 family member heat-shock 70-kDA protein-8 binds to the ubiquitin-like protein monoclonal non-specific suppressor factor β and double knockdown of these factors inhibited RANKL-induced osteoclastogenesis (64). The effects of HSP90 on osteoclastogenesis are more controversial. Whereas, its inhibition with SNX-2112 potently inhibited osteoclast formation (65), the effects of HSP90 inhibition with 17-allylamino-17-demethoxygeldanamycin (17-AAG) on osteoclast formation was shown to be cell-type dependent (66–68).

### S100 Family Proteins

S100 proteins are low molecular weight proteins that belong to the family of calcium binding proteins. Extracellular S100A4 binds to cell surface receptors, such as the RAGE, activating nuclear factor-κB (NF-κB) (69). On mature murine osteoblasts, S100A4 was shown to inhibit mineralization activity and the expression of late-stage osteoblast markers via activation of the NF-κB pathway (70). Additionally, S100A4 has been shown to stimulate osteoclast formation (71). Moreover, although *in vitro* osteoclast cultures with S100A4-deficient bone marrow resulted in more TRAP+ cells compared to wild type cells, the formed osteoclasts were much smaller with less nuclei, underlining the importance for S100A4 in osteoclast formation (72). Finally, binding of S100A4 to extracellular annexins has been shown to regulate the fusogenic activity of osteoclasts (73). The most well-studied S100 proteins in the context of inflammatory bone diseases are S100A8 and S100A9, however data about their direct function on osteoblasts and osteoclasts is rather limited. A previous study showed that stimulation of mature murine osteoclasts with S100A8 enhances their further fusion and resorbing activity *via* binding to TLR4 (74). Another study showed that S100A9 directly stimulates osteoclast formation from monocytes in the context of osteomyelitis in the absence of RANKL (75). However, addition of S100A9 to human monocytes strongly inhibits osteoclast differentiation (76).

### High Mobility Group box Protein 1

High mobility group box protein 1 (HMGB1) is a non-histone nuclear protein that acts as an alarmin extracellularly. TLR2/4/9 and RAGE have been implicated as receptors of extracellular HMGB1. HMGB1 release occurs during tissue injury or microbial invasion via two major pathways, one passive and the other active. Passive release is associated with necrotic cell death, whereas during active release HMGB1 is first shuttled to the cytoplasm, in a JAK-STAT dependent manner, and is thereafter either released into the extracellular space during pyroptosis or alternatively via exocytosis of secretory lysosomes (77, 78). HMGB1 has also been found in EVs. For lymphocytes it is primarily associated with apoptotic vesicles (79), whereas, for macrophages it has also been shown in vesicles released in response to TLR-activation (80). A function for HMGB1 in bone homeostasis has been described, where it can stimulate osteoclastogenesis. HMGB1-RAGE signaling was shown to be important in regulating actin cytoskeleton reorganization, thereby contributing to RANKL-induced and integrin-dependent osteoclastogenesis (81).

### Other Alarmins

Fibronectin also plays a crucial role in the differentiation of osteoblasts (82). Fibronectin is a heterodimeric extracellular matrix glycoprotein that has several cell- and matrix-binding domains (83). Normal human and murine osteoblasts express fibronectin receptors α3β1, α4β1, α5β1, αvβ3, and αvβ5 integrins (84–86). Fibronectin was shown to induce osteoblast differentiation, since perturbation of binding between fibronectin and osteoblasts suppressed nodule formation and maturation, as well as alkaline phosphatase and osteocalcin expression (82). Moreover, fibronectin also displayed pro-survival effect on mature osteoblasts (87). In contrast, fibronectin inhibits the formation of osteoclasts but stimulates the activity of mature osteoclasts *via* nitric oxide and IL-1β-mediated pathways (88). Finally, the antimicrobial peptide of the cathelicidin family LL-37 inhibits osteoclastogenesis by inhibiting the calcineurin activity (89) and the actin-sequestering protein thymosin β4 suppresses osteoclast differentiation (90).

## Summary and Perspective

**Figure 1:**
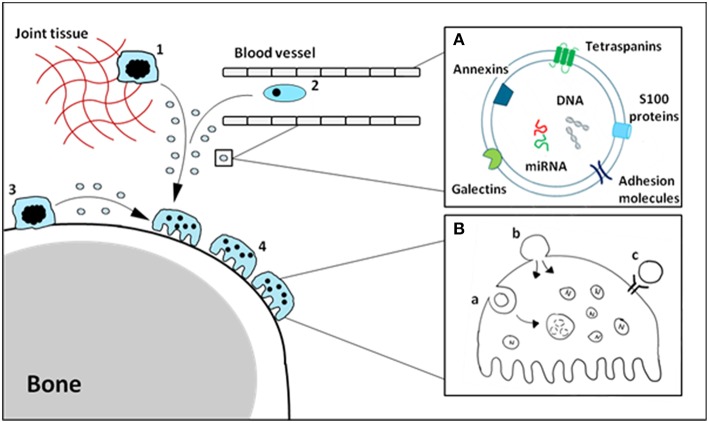
Schematic cartoon of macrophage-derived EVs carrying alarmins impacting osteoclasts. Tissue-resident (1), circulating (2) and osteal macrophages (3) can secrete EVs carrying alarmins **(A)**. These vesicles can interact with bone cells, including osteoclasts (4), in a number of different ways **(B)**. Vesicles can be internalized (a), fuse with the cell membrane (b) or ligands present on the outer membrane of the vesicle can interact with receptors on the cellular membrane (c). The composition and relative quantities of alarmins on macrophage-derived EVs will determine their functional effects.

The function of soluble alarmins has widely been studied, and it is clear there is a profound involvement of alarmins in bone-resident cell differentiation and function. A number of these alarmins have also been identified on EVs derived from monocytes/macrophages, and make up a sizable portion of the vesicle cargo. We hypothesize that vesicle-carried alarmins can have similar effects to soluble alarmins on osteoblast and osteoclast differentiation and function and thereby contribute to bone homeostasis (schematic cartoon in Figure 1).

The composition and relative quantities of alarmins on monocyte/macrophage-derived EVs will ultimately determine their function. Although functional studies are limited, as focus is often on the vesicle as a whole rather than individual proteins carried by the EV, a delicate study by New et al. revealed a critical role of the alarmins Anx5 and S100A9 present on macrophage-derived EVs in microcalcifications in atherosclerotic plaques (36). An additional study, by Nair et al. has shown that LPS stimulated macrophages release microvesicles coated with histones, a different type of alarmin. These histones can interact with TLR4 promoting inflammatory responses (91). On that note, it is important to understand where alarmins are expressed, either on the membrane surface or intravesicularly. Surface bound alarmins can interact with membrane bound receptors on the bone cells, such as TLRs or RAGE, whereas intravesicular alarmins can only interact with intracellular receptors. The mode of uptake is also important in this regard, as a portion of engulfed vesicles are immediately degraded in the lysosome of the recipient cell and therefore will not have to chance to release their alarmins intravesicular.

EVs also contain different molecules such as lipids, polymers of nucleotides, sugars, and other cell metabolites, and when EVs are taken up these molecules will have an impact on the bone cells as well. Nevertheless, alarmins on EVs mediate their first direct contact with bone cells *via* membrane receptor recognition and this interaction could be an effective target to treat bone destruction. Secondly it might be possible to steer either the expression of alarmins in monocytes/macrophages or the EV-loading mechanism toward EVs that possess an anti-inflammatory and bone inducing phenotype. EVs are quite sturdy and can be transportation *via* the circulation, which makes alarmin-EVs important messengers in the local bone remodeling process also when monocytes/macrophages are not in close proximity with the bone cells. An important feature of EVs which enables this distal communication is the ability to integrate with extracellular matrix. Besides carrying alarmins, EVs carry an abundance of adhesion molecules and can bind various matrix molecules allowing interaction with bone (92). This makes EVs uniquely equipped to function as a long-distance alarmin-delivery system to osteoclasts and osteoblasts at the bone site.

Our understanding of extracellular vesicles and alarmins as regulators of bone homeostasis have greatly increased over the past decade, however a role for alarmins on/in extracellular vesicles is often overlooked. Clearly macrophages play a role in bone remodeling and are a source of vesicle-carried alarmins. Future studies should be directed to determine the contribution macrophage-derived EVs have, and identify the alarmin that causes the deregulation of bone homeostasis under inflammatory conditions.

## Outstanding Questions

By reviewing the involvement of alarmins in/on EVs in bone homeostasis, we realized how many questions in this field of research remain unanswered. Below, we highlight a couple of these questions that require further investigation to move this research field forward.

1. How are alarmins associated with EVs, intravesicular or present on the outside of the vesicle membrane? And is there preferential loading for certain types of alarmins?
  Using multiple proteomics approaches, combining proteinase treatment and biotin tagging [method published by Cvjetkovic et al. (49)], it would be possible to delineate which alarmins are present on the outer membrane versus intravesicular.
2. Are alarmins associated with EVs functionally active? And how does this activity relate to soluble alarmins?
  *In vitro* separation of EVs from soluble alarmins derived from macrophages can be difficult, as this heavily depends on the isolation techniques used. If sufficient separation can be achieved, it will be possible to determine how effective each fraction is.
3. In the setting of bone homeostasis, how large is the contribution of EV-associated alarmins compared to soluble alarmins?
  To delineate the contribution of particle-bound versus soluble alarmins we could utilize macrophage-specific knockdown/inhibition of EV-secretion or EV-loading mechanisms, preventing secretion of alarmin-carrying EVs specifically for macrophages.
4. Is there a difference in alarmin content between osteomacs and circulating macrophages? And where are alarmin-carrying EVs produced primarily?
  Comparing the cargo of osteomac- and macrophage-derived EVs using proteomics will shed light on how the EVs differ in alarmin content. It will however remain difficult to trace back the cellular origin of EVs *in vivo* without prior labeling of the producing cells.

## Author Contributions

BP, AC, and MvdB wrote sections of the manuscript. PvL, AT, and FvdL critically revised the manuscript. All authors contributed to manuscript revision, read, and approved the submitted version.

### Conflict of Interest Statement

The authors declare that the research was conducted in the absence of any commercial or financial relationships that could be construed as a potential conflict of interest.

## Abbreviations

DAMP: damage-associated molecular pattern
EV: extracellular vesicle
HSP: heat shock protein
M-CSF: macrophage colony-stimulating factor
MSC: mesenchymal stem cell
NF-κB: activating nuclear factor-κB
RAGE: receptor for advanced glycosylation end products
RANKL: receptor activator of nuclear factor kappa-B ligand
RUNX2: runt-related transcription factor 2
TLR: toll-like receptor.
